# Prevalence of an Insect-Associated Genomic Region in Environmentally Acquired *Burkholderiaceae* Symbionts

**DOI:** 10.1128/aem.02502-21

**Published:** 2022-04-18

**Authors:** Patrick T. Stillson, David A. Baltrus, Alison Ravenscraft

**Affiliations:** a Department of Biology, University of Texas at Arlington, Arlington, Texas, USA; b School of Plant Sciences, University of Arizona, Tucson, Arizona, USA; University of Tartu

**Keywords:** *Burkholderia*, *Caballeronia*, Heteroptera, genomic island, symbiosis

## Abstract

Microbial symbionts are critical for the development and survival of many eukaryotes. Recent research suggests that the genes enabling these relationships can be localized in horizontally transferred regions of microbial genomes termed “symbiotic islands.” Recently, a putative symbiotic island was found that may facilitate symbioses between true bugs and numerous *Burkholderia* species, based on analysis of five *Burkholderia* symbionts. We expanded on this work by exploring the putative island’s prevalence, origin, and association with colonization across the bacterial family *Burkholderiaceae*. We performed a broad comparative analysis of 229 *Burkholderiaceae* genomes, including 8 new genomes of insect- or soil-associated *Burkholderia* sequenced for this study. We detected the region in 23% of the genomes; these were located solely within two *Burkholderia* clades. Our analyses suggested that the contiguous region arose at the common ancestor of plant- and insect-associated *Burkholderia* clades, but the genes themselves are ancestral. Although the region was initially discovered on plasmids and we did detect two likely instances of horizontal transfer within *Burkholderia*, we found that the region is almost always localized to a chromosome and does not possess any of the mobility elements that typify genomic islands. Finally, to attempt to deduce the region’s function, we combined our data with information on several strains’ abilities to colonize the insect’s symbiotic organ. Although the region was associated with improved colonization of the host, this relationship was confounded with, and likely driven by, *Burkholderia* clade membership. These findings advance our understanding of the genomic underpinnings of a widespread insect-microbe symbiosis.

**IMPORTANCE** Many plants and animals form intricate associations with bacteria. These pairings can be mediated by genomic islands, contiguous regions containing numerous genes with cohesive functionality. Pathogen-associated islands are well described, but recent evidence suggests that mutualistic islands, which benefit both host and symbiont, may also be common. Recently, a putative symbiosis island was found in *Burkholderia* symbionts of insects. We determined that this genomic region is located in only two clades of *Burkholderia* (the plant- and insect-associated species) and that although it has undergone horizontal transfer, it is most likely a symbiosis-associated region rather than a true island. This region is associated with improved host colonization, although this is may be due to specific *Burkholderia* clades’ abilities to colonize rather than presence of the region. By studying the genomic basis of the insect-*Burkholderia* symbiosis, we can better understand how mutualisms evolve in animals.

## INTRODUCTION

Genomic islands are clusters of genes in bacterial and archaeal genomes which usually show signs of horizontal transfer between lineages and which often encode proteins and pathways that help microbes thrive in specialized niches (1). The first and best-studied examples are pathogenicity islands, which encode machinery for pathogenesis, virulence, or antibiotic resistance and contribute to the rapid evolution of pathogens (1). The increasing availability of bacterial genome sequences highlights the prevalence and importance of genomic islands across all bacteria, not just pathogens (1, 2). A few nonpathogenic genomic islands have been shown to enable a symbiotic lifestyle by facilitating bacterial mutualisms with multicellular hosts. This includes enabling, or improving, symbiont colonization of the host, survival within the host, and/or production of beneficial compounds that underpin the relationship (1).

To date, the best-studied symbiotic island is found in the legume-nodulating rhizobial symbiont Mesorhizobium loti (3, 4). In the legume-M. loti symbiosis, island genes are associated with root nodule formation and nitrogen fixation, core functions for a relationship which involves provisioning of biologically available nitrogen to the host plant (5). Notably, although the island is located on the chromosome, it undergoes frequent horizontal transfer, which can result in transmission of the rhizobial lifestyle to previously nonsymbiotic bacteria (6). Recent studies have found that horizontal transfer of the genomic island carrying nodule formation and nitrogen fixation genes is a common feature of legume-rhizobium symbioses, occurring within and between many rhizobial genera across the alpha- and betaproteobacterial classes (5).

Only a few other symbiosis-enabling islands have been documented outside legume-rhizobium symbioses. Some entomopathogenic nematodes rely on *Photorhabdus* bacteria to aid in parasitizing their insect prey; both the genes *Photorhabdus* uses to associate with its host and many of the virulence factors it uses to kill insect prey are carried and encoded, respectively, on genomic islands (7). Similarly, in the squid-*Vibrio* symbiosis, different strains of Vibrio fischeri will compete for dominance in the host’s light organ using a type VI secretion system that is encoded within a genomic island (8). However, unlike the legume-rhizobium island, these islands either carry genes that aid symbiont survival within the host or encode pathogenic functions that have been co-opted to benefit the host, rather than encoding services that are specifically beneficial for the host. To date, the rhizobial symbiosis remains the best, and arguably only, example of a genomic island which encodes services that are expressly beneficial for the host.

Recently, a putative genomic island was detected that appears to be associated with the symbiotic relationship between hundreds of species of true bugs in the superfamilies Coreoidea, Lygaeoidea, and Pyrrhocoroidea (Hemiptera: Heteroptera: Pentatomomorpha) and bacteria in the genus *Burkholderia sensu lato* (referred to here as *Burkholderia*) (9–11). In this symbiosis, insects house *Burkholderia* cells within sac-like outgrowths called “crypts” in a specialized section of the midgut, the M4. The insect digests excess *Burkholderia* cells to obtain various nutrients, including sugars, fatty acids, essential amino acids, and B vitamins (12, 13). *Burkholderia* also appears to play an important role in recycling the insect’s nitrogenous waste (12). Surprisingly, given the nutritional importance of this association, young nymphs must acquire *Burkholderia* from the environment each generation or else suffer high mortality, developmental delays, and reduced body size (14). Colonization success of the M4 crypts by environmental *Burkholderia* varies between species and strains, with the most beneficial symbionts fully utilizing the space within the crypts, leading to an enlarged, opaque M4, while poor symbionts do not readily colonize the crypts and the M4 remains translucent and shrunken (15). Colonization of the M4 is coordinated by both host factors, such as a selective constricted region anterior to the M4 which is blocked by mucus laced with antimicrobial peptides (16–19), and bacterial adaptations to overcome these barriers (17, 19). These mechanisms prevent most non-*Burkholderia* species from entering the M4 region of the gut, but some non-*Burkholderia* species can colonize the crypts and act as symbionts, such as *Pandoraea*, although these alternative symbionts have problems fully colonizing the M4 compared to *Burkholderia* (15).

A putative symbiotic island was recently found in two different clades of *Burkholderia* (9), the SBE clade (stink bug-associated beneficial and environmental clade, recently elevated to the genus *Caballeronia*) (20) and the iPBE clade (an insect-associated clade within the plant beneficial and environmental group PBE; PBE was recently elevated to the genus *Paraburkholderia*) (21). This contiguous 38-gene region was proposed to be a symbiotic island by Takeshita and Kikuchi because it was conserved across five insect-associated *Burkholderia* genomes and more than half of its genes were upregulated during symbiosis (9). In all five of the genomes analyzed the region was located on a plasmid, suggesting the potential for horizontal transfer, similar to the dynamics observed for the rhizobial symbiotic island. Notably, this genomic region includes a cluster of four genes used to synthesize a pyrroloquinoline-quinone (PQQ) cofactor as well as a PQQ-dependent alcohol dehydrogenase (PQQ-ADH) enzyme (9). In *Drosophila*, the production of PQQ and PQQ-ADH by gut bacteria modulates insulin signaling and thus regulates host metabolic homeostasis, ultimately controlling host development, body size, and energy metabolism (22). This contiguous 38-gene region likely plays an important role in bug-*Burkholderia* symbiosis, but since the initial analysis focused on five genomes representing the SBE and iPBE subclades, the prevalence, origin, and functional effects of the region across the extensive diversity of *Burkholderia* remain unknown.

We searched for the symbiosis-associated region across 221 genomes downloaded from GenBank (October 2020) representing the entire *Burkholderiaceae* family, plus 8 new genomes which we generated from bug symbionts collected within the United States. We expected that the region would be found only in bacterial species shown to be capable of participating in bug-*Burkholderia* symbiosis. We found that the contiguous region was frequently present in members of the SBE clade (22/30 genomes), PBE clade (29/61 genomes), and *Trinickia* spp. (formerly *Paraburkholderia* [23]; 2/4 genomes) but lacking entirely in the pathogenic “Burkholderia cepacia complex and Burkholderia pseudomallei” clade (BCC&P, or *Burkholderia sensu stricto*; 0/34 genomes). Upon further investigation, all the genes detected within the contiguous genomic region were detected within other species of the *Burkholderiales* order, suggesting a more ancestral origin of the genomic region. These data suggest that all genes in the region existed in the common ancestor of *Burkholderia sensu lato* and subsequently underwent several loss events within the genus. Phylogenetic analyses suggest that this region has a low degree of synteny across genomes, and although the region was almost always localized to a chromosome (50/53 genomes) instead of a plasmid, as originally observed, we found evidence of occasional horizontal transfer of the region within *Burkholderia*.

## RESULTS

### Genome assemblies.

Because there were only five complete genomes available from GenBank that were collected from true bugs, and they were derived from Riptortus pedestris (Alydidae) and other Asian true bugs (9, 24–26), we constructed eight new genomes, with seven strains isolated from true bugs in North America and one strain isolated from soil underneath a population of true bugs. Five of our genomes were complete and circularized. The other three genomes were mostly complete, but some of the chromosomes or plasmids were not circularized. Genomes consisted of 3 to 5 chromosomes and 2 to 6 plasmids (Table 1).

**TABLE 1:** Genome species identifications and summary statistics

Parameter	A33_M4_a	BHJ32i	Lep1A1	Lep1P3	SL2Y3	SMT4a	Sq4a	TF1N1
Accession no.	CP084283–CP084289	CP084279–CP084282	CP084271–CP084278	CP084265–CP084270	CP084260–CP084264	CP084255–CP084259	CP084250–CP084254	CP084626–CP084634
Clade	SBE	*Cupriavidus*	SBE	SBE	SBE	PBE	SBE	SBE
Host family	Coreidae	Alydidae	Coreidae	Coreidae	Berytidae		Coreidae	Berytidae
Host species	*Anasa. tristis*	*Alydus tomentosus*	*Leptoglossus zonatus*	*Leptoglossus zonatus*	*Jalysus wickhami*	Soil	*Anasa tristis*	*Jalysus wickhami*
Location	Georgia	Georgia	California	California	Arizona	Georgia	Georgia	North Carolina
Closest species^*a*^	Caballeronia zhejiangensis (99.80)	Cupriavidus pauculus (99.00)	Caballeronia grimmiae (97.00)	Caballeronia concitans (86.00)	Caballeronia concitans (86.00)	Paraburkholderia sediminicola (99.20)	Caballeronia concitans (86.00)	Caballeronia concitans (84.00)
Genome size (Mb)	7.41	6.17	6.65	6.18	5.51	7.21	6.01	7.07
GC content (%)	63.0	64.1	62.8	63.7	64.1	63.6	63.9	61.6
No. of chromosomes	3	2	3	3	3	3	3	3
No. of incomplete chromosomes	0	1	0	0	0	2	0	1
No. of plasmids	4	2	5	3	2	3	2	5
No. of incomplete plasmids	0	0	0	0	0	3	0	1
No. of CDSs^*b*^^,^^*c*^	6,927	5,557	6,199	5,764	5,024	6,410	5,560	6,457
Coding ratio (%)^*b*^	87.4	88.1	86.1	86.8	87.2	85.5	86.7	87.3
Avg protein length (aa)^*b*^^,^^*d*^	311.7	326.4	308.1	310.3	318.7	320.5	312.5	318.6
No. of rRNAs^*b*^	12	12	15	10	10	14	10	10
No. of tRNAs^*b*^	69	69	68	64	66	72	69	66

a Values in parentheses indicate percent ANI to the listed species. If over 94%, they are the same species (68).

b Calculated with DFAST (56).

c CDSs, coding DNA sequences.

d aa, amino acids.

### Phylogenetic analysis and species identification.

**FIG 1:**
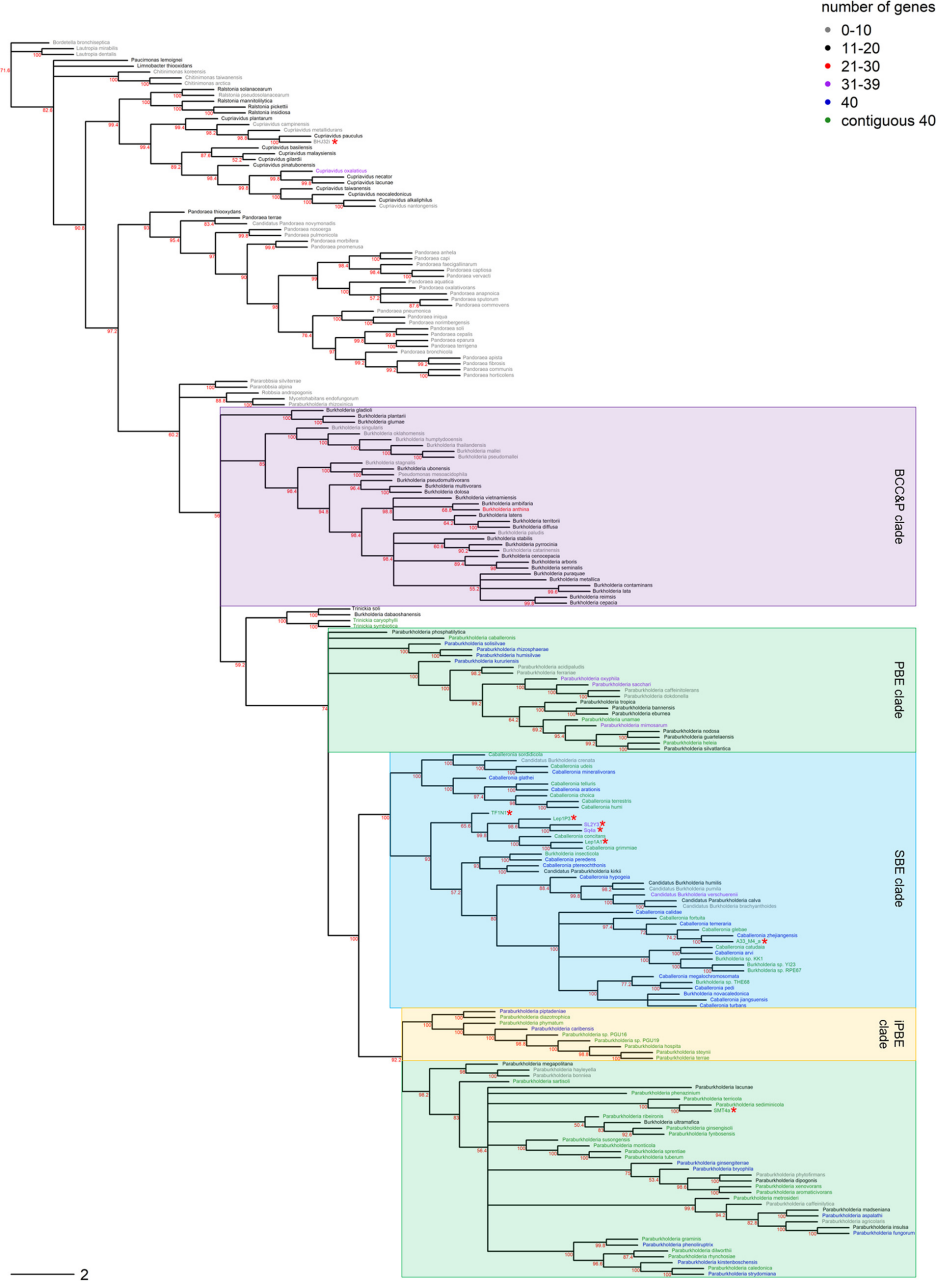
*Burkholderiaceae* whole-genome phylogeny. The phylogeny of *Burkholderiaceae* was constructed using Realphy to compare whole genomes. Known clades within the *Burkholderia* genus are highlighted, and the newly sequenced genomes for this study are marked with a red asterisk. Text color indicates the number of genes from the contiguous genomic region which a species possessed. The tree was rooted with outgroup Bordetella bronchiseptica. Node support values were calculated using rapid bootstrapping, and nodes with less than 50% support have been collapsed. Scale bar indicates substitutions per site.

To determine the placement of our genomes within *Burkholderiaceae*, a whole-genome phylogeny was constructed. We recovered the known clades of *Burkholderia* (Fig. 1). Six of our eight isolates were identified as members of the SBE clade, one was a member of the PBE clade, and one, the soil isolate, belonged to the genus *Cupriavidus*. Average nucleotide identity (ANI) values indicated that four of our isolates belonged to the same species as those located within their terminal clades (Table 1). Specifically, we identified Lep1A1 as Caballeronia grimmiae (ANI, 97.00%), SMT4a as Paraburkholderia sediminicola (ANI, 99.20%), A33_M4_a as Caballeronia zhejiangensis (ANI, 99.80%), and BHJ32i as Cupriavidus pauculus (ANI, 99.00%). Four of the isolates we sequenced were not members of any named species within the genomes found in GenBank; these were most closely related to Caballeronia concitans, which shared 86.00% average nucleotide identity with Lep1P3, SL2Y3, and Sq4a and 84.00% identity to TF1N1. The closest relative of SL2Y3 was Sq4a (ANI, 94.60%), although these isolates were collected from insects located almost 2,500 km apart in Arizona and Georgia, respectively.

Ancestral state reconstruction was used to determine when the contiguous region likely arose within *Burkholderiaceae*. There is an 85% chance that the putative island arose at the division of SBE and PBE *Burkholderia* from the rest of *Burkholderia sensu lato* and a 45% chance that it arose at the common ancestor of PBE, SBE, and *Trinickia* after the divergence of the BCC&P clade.

### Genome comparisons.

To test the hypothesis that all symbiotic strains of *Burkholderia* must possess the contiguous symbiosis-associated region, we first searched for the PQQ gene cluster, as these island-associated genes were found to be upregulated during symbiosis (9). Of the 229 genomes analyzed, 84 lacked the PQQ gene cluster (see Table S1 in the supplemental material). The 145 genomes containing the PQQ gene cluster were further analyzed using a Mauve alignment to find the contiguous genomic region. There were 92 genomes that contained the PQQ gene cluster but did not possess the entire region. Additionally, there were 17 genomes in which the region was divided between two or more contigs (from unassembled genomes) and therefore could not be assessed for completeness. Overall, a total of 53 out of 229 genomes (of which 212 were analyzable for genomic region completeness) contained the contiguous region, including 22/30 SBE species, 29/61 PBE species (of which it was found in 7/8 iPBE species), and 2/4 *Trinickia* spp. This region was not found in any of the BCC&P genomes or outside *Burkholderia*. The region was located on chromosome 2 (38% of genomes), chromosome 3 (4%), plasmid 2 (4%), and plasmid 1 (2%). Chromosomal placement could not be determined for the contig-based assemblies (53% of genomes containing the entire genomic region).

Takeshita and Kikuchi’s initial report of the putative symbiotic island identified 38 contiguous conserved genes, of which 24 were upregulated during symbiosis and 14 encoded hypothetical proteins of unknown function (7 of which were upregulated) (9). We identified 40 conserved genes, adding the 4a-hydroxytetrahydrobiopterin dehydratase and fructose-1,6-bisphosphatase genes to those previously identified. We also determined the putative functions of 12 of the 14 coding regions originally characterized as hypothetical proteins by searching for sequence similarity using BLASTX on the GenBank database and by comparing unannotated genes within one clade (e.g., SBE) to their annotated orthologs in another clade (e.g., PBE). The hypothetical proteins were (5-formylfuran-3-yl)methyl phosphate synthase, aspartate kinase, two different ATP-grasp domain-containing proteins, coenzyme PQQ synthesis protein E, *c*-type cytochrome, dihydroneopterin aldolase, DUF447 family protein, formylmethanofuran dehydrogenase subunit B, histidine kinase, phosphoribosylformimino-5-aminoimidazole carboxamide ribotide isomerase, and response regulator transcription factor. Additionally, a fragment of the pyrroloquinoline quinone precursor peptide PQQA was detected. The full list of genes present in each species’ symbiosis-associated region and the genes’ expression status during symbiosis (as measured by Takeshita and Kikuchi [9]) is provided in Table S2.

Of the newly identified proteins, the pyrroloquinoline quinone precursor peptide PQQA did not code for the whole protein and instead was a fragment of approximately 100 bp. Due to the small size, the annotation was found in only 42 of the 53 islands, but when the unannotated regions were searched for on NCBI using BLASTX, the PQQA fragment was found in the remaining 11 genomes with complete islands.

In general, gene content and order within the symbiosis-associated region, across all 53 genomes, were similar to those reported by Takeshita et al. (26), except for the presence of a highly variable region within 46 isolates’ islands. This unconserved region contained two to five coding regions which encoded fructose-1,6-bisphosphate aldolase (81% of genomes), transketolase (77%), a hypothetical protein (40%), thioredoxin family protein (26%), ribulose-phosphate 3-epimerase (13%), twitching motility protein PilT (11%), type II toxin-antitoxin system VapC family toxin (8%), ankyrin repeat domain-containing protein (2%), AraC family transcriptional regulator (2%), AzlC family ABC transporter permease (2%), AzlD domain-containing protein (2%), cytochrome *c*_4_ (2%), muraminidase (2%), helix-turn-helix transcriptional regulator (2%), PAAR domain-containing protein (2%), PQQ-dependent dehydrogenase, methanol/ethanol family (2%), redoxin domain-containing protein (2%), type II toxin-antitoxin system HipA family toxin (2%), and type VI secretion protein Vgr (2%). Seven of the analyzed islands did not contain this region; five were in the iPBE clade, while the other two, SL2Y3 and Sq4a, were SBE species. Interestingly, SL2Y3 and Sq4a were also missing an additional 6 genes that were present in all 51 other species’ putative islands (Table S2).

A follow-up analysis of the 176 *Burkholderiaceae* species that did not possess the entire genomic region revealed all had orthologous island-associated genes in their genomes, detected using BLASTX. Of these, 28 species had all 40 of the genes from the symbiotic genomic region but these were not contiguous, 6 had 20 to 39 of the genes in a noncontiguous placement, and 142 had fewer than 20 genes from the symbiotic region (Table S3). In addition, two of the genes missing from the contiguous region in SL2Y3 and Sq4a had orthologous genes located elsewhere in those genomes, specifically, LysR family transcriptional regulator and fructose-1,6-bisphosphatase class 1. Orthologs of the 40 genes were also detected in related families within the order *Burkholderiales*, including *Alcaligenaceae*, *Comamonadaceae*, *Ideonella*, and *Oxalobacteraceae*.

### Horizontal transfer of the bug-*Burkholderia* genomic region.

**FIG 2:**
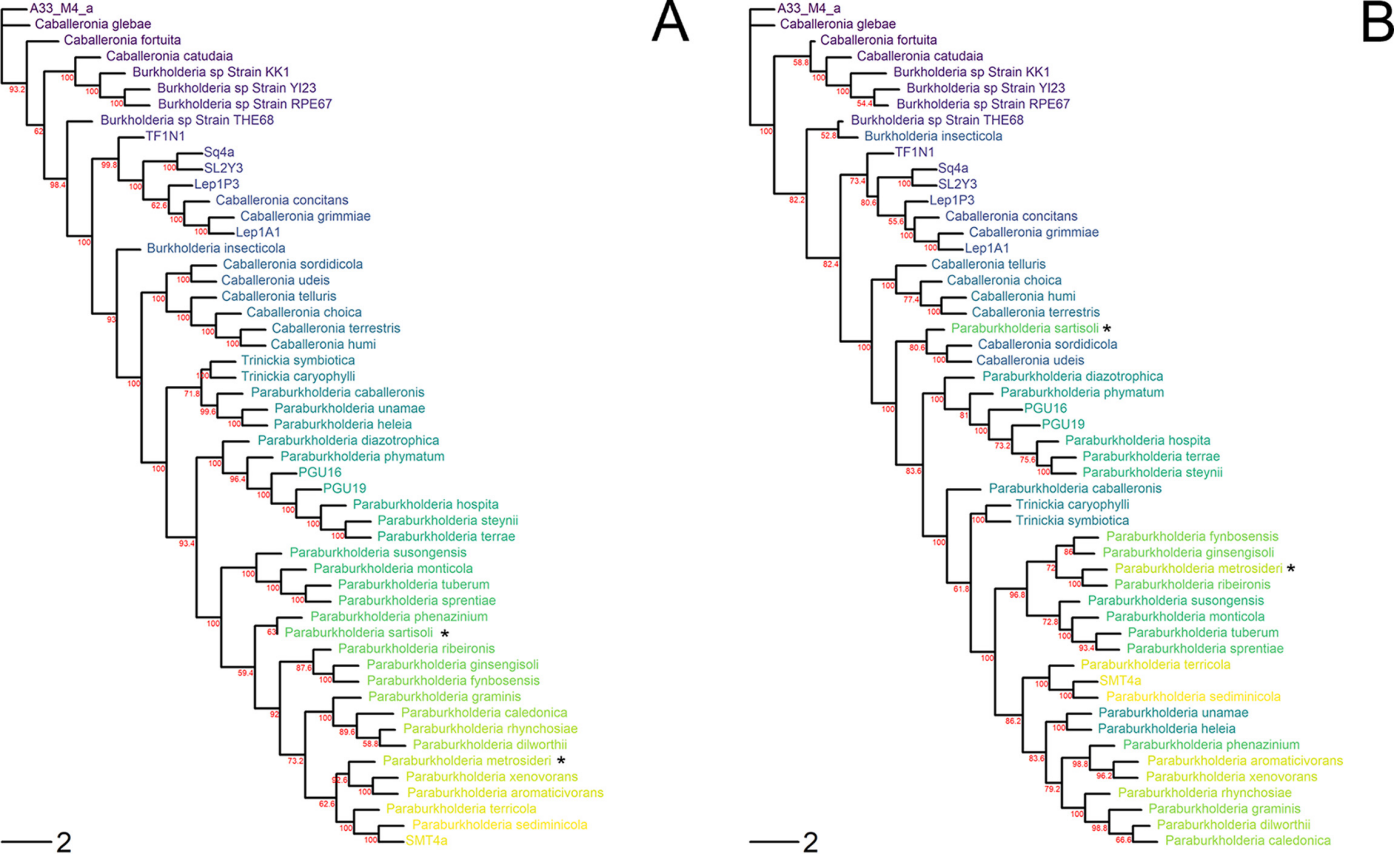
Comparison of whole-genome phylogeny to an upregulated gene from the contiguous genomic region. Phylogenetic arrangement of species with the contiguous genomic region based on whole genomes after removal of the 40-gene region (A) and sigma-54-dependent Fis family transcriptional regulator (B) from the genomic region is shown. (The phylogenies of three other upregulated island genes followed a pattern similar to that of the sigma-54-dependent Fis transcriptional regulator; see Fig. S1.) Trees were rooted with species A33_M4_a for consistency, as no outgroup was available. Species names in the phylogenies were color-coded based on the order listed in the whole-genome phylogeny to improve visualization of incongruencies. The black asterisks indicate potential horizontal transfer events. Node support values were calculated using rapid bootstrapping, and nodes with less than 50% support have been collapsed. Scale bar indicates substitutions per site.

To identify instances of horizontal transfer of the genomic region within *Burkholderia*, we compared the whole-genome phylogeny (with the contiguous 40-gene region removed) to the phylogenies of four separate genes from the genomic region that are upregulated during symbiosis (Fig. 2 and Fig. S1). Finding the same species or clade in different positions between the whole-genome phylogeny versus the gene phylogenies would suggest a potential horizontal transfer event. Based on the incongruencies detected between the phylogenies, Paraburkholderia sartisoli and Paraburkholderia metrosideri may have experienced horizontal transfer events. These species were found in different clades within the upregulated gene phylogenies compared to the whole-genome phylogeny. Paraburkholderia sartisoli had incongruencies in 2/4 phylogenies (Fig. S1). The terminal clade consistently aligned with Caballeronia sordidicola and Caballeronia udeis, suggesting that the symbiosis-associated region may have been transferred from a *Caballeronia* ancestor of these species. Paraburkholderia metrosideri was observed in a different subclade in all 4 of the gene phylogenies (Fig. S1) and was consistently located within the same terminal clade as Paraburkholderia ribeironis, although these two species are in different subclades in the whole-genome phylogeny (Fig. 1). Interestingly, *P. metrosideri* is the only species in its subclade that has the contiguous genomic region. This suggests that the subclade lost the region and *P. metrosideri* may have received a new copy of these genes from *P. ribeironis*.

Although we found potential interspecies horizontal transfer events, across all 53 species with the putative island, the regions flanking the island did not contain any mobility elements, integrases, or direct flanking repeats that could drive horizontal transfer. Phage elements were detected on 17/53 chromosomes (or contigs) that contained the island, but only 3/53 contained any phage elements within 50,000 bp of the island and none were closer than 30,000 bp. Due to this distance, phage elements likely have not contributed to horizontal transmission. It is possible that the island could be transferred via conjugation if it is located on a plasmid, but we were unable to determine the genomic location of the putative island for the two likely lateral transfer events because these involved contig-based assemblies.

### Testing necessity of the genomic region for bug colonization.

**FIG 3:**
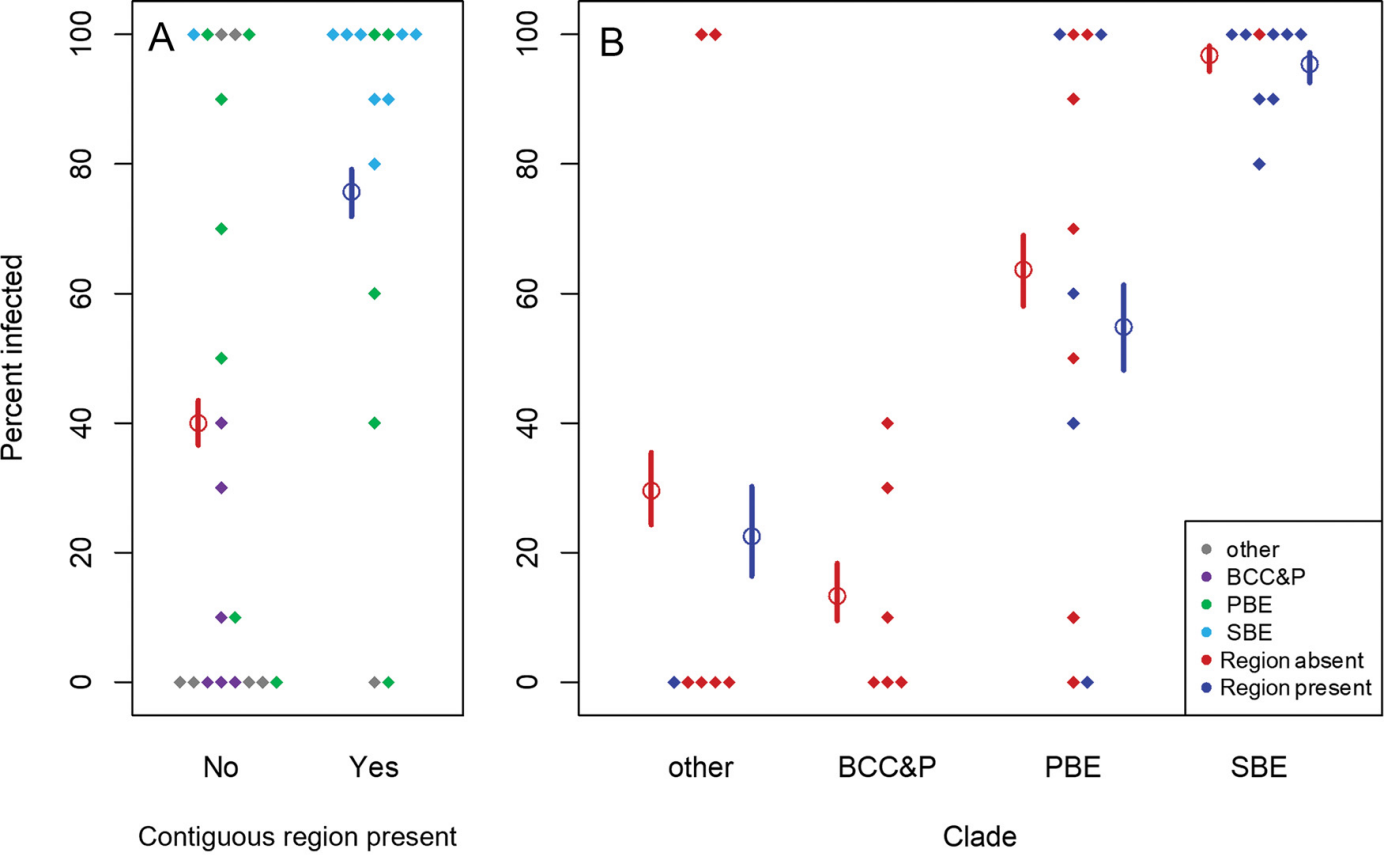
Colonization success by presence of the contiguous region and clade membership. M4 colonization data from Itoh et al. (15) were compared between species with and without the contiguous genomic region, and among *Burkholderiaceae* clades. Diamonds represent the percentage of insects that were PCR positive for the colonizing bacterium after 10 individual insects were exposed to each bacterial species. Hollow circles indicate model-predicted means for a given category, and lines indicate standard errors. (Means were not predicted for categories with no data points. Because standard errors were calculated from the model’s pooled variance, categories with a single data point do still have error bars.) (A) Comparison between species without and with the genomic region: when clade is not included as a predictor, the region is associated with increased colonization success. Diamonds are colored based on the known clades within the *Burkholderia* genus: purple for BCC&P, green for PBE, light blue for SBE, and gray representing other species outside of these clades. Model predictions are colored based on possession of the region: red for absent and dark blue for present. These predictions are based solely on the presence or absence of the genomic region. (B) Comparison among clades: presence of the contiguous genomic region is not associated with colonization success after controlling for clade membership. Data points and model predictions are colored based on possession of the region, as in panel A. These predictions are based both on genomic region presence and on clade membership.

We were able to determine the presence or absence of the contiguous genomic region in 34 *Burkholderiaceae* species which Itoh and colleagues tested for M4 colonization success (15). They infected *R. pedestris* with different *Burkholderiaceae* species and then measured host infection status by performing diagnostic PCR on 10 individuals to determine the overall infectivity of each symbiont strain. Of these, 20 *Burkholderia* species lacked the genomic region and 14 possessed it. Before accounting for clade membership, species that possessed the region were 4.7 times more likely to colonize the insect M4 organ (χ^2^ = 43.9, *df* = 1, and *P* < 0.001) (Fig. 3A). However, the presence of the contiguous region was confounded with clade membership. For example, within the SBE clade only one species (Caballeronia glathei) lacked the genomic region, although all nine species (including *C. glathei*) exhibited high colonization success (80 to 100% infection rate) (Fig. 3). Within the PBE clade, seven species lacked the contiguous region and five species had the region, and there was no correlation between the presence of the genomic region and colonization success (χ^2^ = 0, *df* = 1, and *P* = 1). When we included both presence of the region and clade as predictors, only clade was correlated with colonization success (clade, χ^2^ = 100.5, *df* = 3, and *P* < 0.001; island, χ^2^ = 1.2, *df* = 1, and *P* = 0.3).

Twenty-one additional species contained island genes but lacked the entire contiguous region; of these, only 6 species had high colonization success (>50%): *C. glathei*, Caballeronia megalochromosomata, Paraburkholderia fungorum, and Paraburkholderia tropica, with 100% colonization rates, Paraburkholderia kururiensis, with 90% colonization, and Paraburkholderia caribensis, with 70% colonization. Of these species, all but *P. tropica* possessed all 40 of the genes found in the contiguous region, while *P. tropica* had 14/40 genes. Two additional species had high colonization success—Pandoraea norimbergensis (100% success) and Pandoraea oxalativorans (100%)—but possessed only seven genes from the region. The success of these species suggests that genes found elsewhere in the genome likely contribute to colonization success.

## DISCUSSION

Here, we report that a recently discovered contiguous genomic region associated with bug-*Burkholderia* symbiosis is distributed throughout the SBE (*Caballeronia*) and PBE (*Paraburkholderia*) clades of *Burkholderia*, as well as in *Trinickia*. In contrast, the contiguous region is absent from all other genera with published genomes in the family *Burkholderiaceae* (including the pathogenic BCC&P clade, or *Burkholderia sensu stricto*). The contiguous arrangement of this genomic region likely arose in the common ancestor of PBE and SBE *Burkholderia* and *Trinickia* and has since undergone several loss events. Based on the analysis of 53 genomes that contained the entire region, we identified two additional conserved genes to add to the 38 previously identified by Takeshita et al. (9) and determined the putative function of 12 of the 14 genes originally characterized as hypothetical proteins. We also identified a highly variable unconserved region of 2 to 5 coding sequences found within the putative island of most species. In contrast to the rhizobial symbiotic island, horizontal transfer of the putative bug-*Burkholderia* island appears to be rare within *Burkholderiaceae*: we identified only two possible instances of horizontal transfer. The region was almost always located on a chromosome and was not associated with any mobility elements, which suggests that it may not be a genomic island after all. Finally, we found that this region was not correlated with colonization of the insect’s M4 organ after accounting for differences among bacterial clades. Whether the region encodes other functions that benefit the insect remains an open question.

### Phylogenetic distribution of the contiguous genomic region within *Burkholderiaceae*.

The whole-genome phylogeny presented here is the largest analysis of *Burkholderiaceae* to date, providing predictions of evolutionary relationships within this family. This tree indicates that the contiguous genomic region is found only within SBE and PBE *Burkholderia* and is absent in other members of *Burkholderiaceae*. Interestingly, this includes the genera *Pandoraea* and *Cupriavidus*, even though these are capable of colonizing the M4 (15, 27).

Within the *Burkholderia* genus, the phylogeny revealed that the BCC&P clade is ancestral to the PBE and SBE clades. As the first whole-genome phylogeny of the *Burkholderia* genus, this provides the best hypothesis of relationships within *Burkholderia* to date and confirms a recent phylogenetic analysis of *Burkholderia* based on 21 marker genes (15). Additionally, monophyly of the three subclades of *Burkholderia* supports the proposed splitting of the genus into the genera *Burkholderia sensu stricto* (BCC&P), *Paraburkholderia* (PBE), and *Caballeronia* (SBE) (20, 21, 23).

Within the set of genomes for which we could assess the presence of the contiguous genomic region, it was found within the majority of SBE species (73%), half of PBE species (48%), most of the insect-associated PBE (iPBE; 88%), and half of *Trinickia* species (50%). The region was entirely absent from the BCC&P clade. Therefore, the region was most prevalent in clades known to associate with insects (particularly SBE and iPBE), but contrary to our expectations, it was not universally present within these clades.

Via a BLASTX search, we found orthologs to all 40 genes located within the genomic region across *Burkholderia sensu lato*, *Burkholderiaceae*, and even within other families in the order *Burkholderiales*, indicating that these genes are ancestral and were not derived within the *Burkholderia* genus. Despite this, the contiguous arrangement is unique to species found within *Trinickia* and the PBE and SBE clades.

### Horizontal transfer of the bug-associated genomic region is uncommon.

Genomic islands are characterized by horizontal transfer between lineages (1, 28). This is the case for rhizobial symbionts, which appear to frequently transfer their symbiotic island between species and genera (5). Horizontal transfer of the bug-associated genomic region also appears to occur, although infrequently. Incongruencies between the whole-genome phylogeny and island gene phylogenies suggest two possible horizontal transfer events for the contiguous genomic region among 53 species. There was also evidence of island mobility within genomes, as this region was found on all three chromosomes and even detected on plasmids. The genomic region therefore appears to be more mobile within genomes than between species. The low rate of interspecies horizontal transfer may result from loss or lack of island-associated mobility elements. Genomic islands are often flanked by mobility elements or located on plasmids, facilitating horizontal transfer of the pathogenic or symbiotic lifestyle (1, 5). However, after a genomic island is acquired by a new microbe, this transfer machinery is often lost via reductive evolution, decreasing the probability of future horizontal transfer events (1, 29). This may have occurred in *Burkholderia*. However, the lack of evidence for flanking mobility elements in the more basal lineages suggests that mobility elements may never have accompanied the bug-associated region, and other processes with rarer probabilities of lateral transfer, like recombination, may have driven transfer events.

In fact, the absence of detectable mobility elements and the ancestral origin of genes comprising this region within *Burkholderiales* raise the question of whether this symbiosis-associated region should be considered a genomic island. Although Takeshita and Kikuchi first detected the putative island on plasmids (9), our study indicates that it is predominantly located on chromosomes. However, we cannot reject the possibility that flanking mobility elements have been lost over evolutionary time. We do find that the region is always found in the same order, even when it moves between chromosomes, and we observed two likely horizontal transmission events among 53 genomes. More research will need to be conducted to determine what factors are associated with the observed horizontal transfer events.

Interestingly, in rhizobial *Burkholderia*, horizontal transfer of the legume symbiotic island occurs frequently in South African species (30) but is rare in South American species, similar to the case with the bug-*Burkholderia* genomic region (31). This may suggest that *Burkholderia* might be less biologically inclined to horizontally transfer genetic material in certain habitats. Interestingly, unlike for alphaproteobacterial rhizobia like the model Mesorhizobium loti, in rhizobial *Burkholderia*, symbiotic nodulation genes are located on a plasmid (30). Comparison of the rhizobial symbiotic island and the bug-*Burkholderia* genomic region could therefore provide insight into many factors that govern horizontal transfer of symbiotic lifestyles, addressing questions such as the following: why are some bacterial clades more likely to participate in horizontal transfer than others, and what environmental factors and what features of genomic regions (e.g., location within the genome or presence of mobility elements) influence rates of horizontal transfer?

### Putative function of newly identified genes.

We identified two additional conserved genes within the symbiosis-associated region (encoding 4a-hydroxytetrahydrobiopterin dehydratase and fructose-1,6-bisphosphatase) in addition to the 38 previously identified by Takeshita and Kikuchi (9). The dehydratase is primarily involved in aromatic amino acid biosynthesis (32), while fructose-1,6-bisphosphatase is utilized in many metabolic processes (33). Both genes are widespread across animals, plants, and microbes and are not necessarily related to symbiosis.

We additionally determined the putative function of 12 of the 14 regions originally characterized as hypothetical proteins by Takeshita and Kikuchi (9). Seven of these were not upregulated (or regulatory status was not reported) during symbiosis and received only general functional predictions, so their relevance for bug symbiosis, if any, is unclear. Five of the newly annotated genes were upregulated during symbiosis (9), including the genes for aspartate kinase, *c*-type cytochrome, coenzyme PQQ synthesis protein E, dihydroneopterin aldolase, and response regulator transcription factor. Three of these upregulated genes had functions potentially relevant for symbiosis. Aspartate kinases are only found in microbes and plants and are used in the biosynthesis of four amino acids, including aspartate, methionine, lysine, and threonine, with the last three being essential for animals (34). *Burkholderia* might therefore provide a supplementary source of these amino acids for insect hosts, as has been observed in other hemipteran symbioses (35, 36). Dihydroneopterin aldolase is used in synthesis of folate (37), a compound needed by animals for proper development (38).

The third gene, encoding coenzyme PQQ synthesis protein E, belongs to the PQQ biosynthesis pathway (12). Many genes involved in this pathway are upregulated in symbiotic *Burkholderia*, specifically, PQQB, PQQC, PQQD, and PQQE, which are all present in the symbiosis-associated region (12). When *Drosophila*’s gut microbe Acetobacter pomorum lacks the PQQ biosynthesis pathway, the host exhibits slowed growth, smaller body size, and problems maintaining insulin-associated metabolic homeostasis (22). This suggests that the symbiont’s PQQ genes could be involved in host metabolism and homeostasis, although this has not yet been studied in the bug-*Burkholderia* system.

Along with these newly identified genes, we detected a fragment of the pyrroloquinoline quinone precursor peptide PQQA not only in symbiont species but across all *Burkholderia* species, indicating that degradation of this gene began prior to the evolution of bug association. Other bacterial species, like Methylobacterium extorquens, do not require PQQA for pyrroloquinoline quinone biosynthesis (39). This PQQA fragment, therefore, is most likely unnecessary for the bug-*Burkholderia* symbiosis.

### Interspecies variation in genomic region composition.

We identified a highly variable section of 2 to 5 coding regions within the putative island of most (46 out of 53) species. Of the species lacking this variable section, two were SBE strains and five were from the iPBE clade. Given the highly variable nature, Takeshita and Kikuchi were unable to analyze whether any of the included genes are up- or downregulated during symbiosis, and we were not able to assess whether these genes are associated with colonization success (9). However, the high variation in gene content suggests that the variable section is likely not associated with bug symbiosis.

Of the 53 strains with the symbiosis-associated region, SL2Y3 and Sq4a were the only SBE strains that lacked the variable unconserved section. These strains were unique in that they were also missing six additional genes that were present in all other islands, including two genes which Takeshita et al. found to be upregulated during symbiosis: the genes encoding the LysR family transcriptional regulator (orthologs were present elsewhere in the genome) and ribulose bisphosphate carboxylase large chain (25). One might expect that strains lacking these genes are inferior symbionts. M4 colonization data were not available for these strains, which appear to be representatives of one unnamed species or two closely related unnamed sister species. However, surprisingly, we found that insects colonized with SL2Y3 or Sq4a are comparable to insects infected with other strains in terms of development time and mass at adulthood (27, 40). This indicates that not all genes in the genomic region—and not even all genes found to be upregulated during symbiosis—are required for effective symbiosis. Similarly, the set of genes that were upregulated in symbiotic *Snodgrassella* bacteria when they colonized bee guts largely did not overlap the set of genes found to be essential for establishment of the symbiosis via transposon sequencing (41). These findings indicate that not all genes upregulated during symbiosis necessarily contribute to colonization success or provide benefits to the host.

### Increased colonization success could be driven by island presence or by clade membership.

Although we found that species with the genomic region were more likely to colonize the insect M4, this relationship disappeared when we accounted for differences in colonization success among *Burkholderia* clades. We did not have sufficient statistical power to distinguish whether clade affiliation or presence of the genomic region determines colonization success because these attributes were highly correlated. However, it seems likely that colonization success is driven by differences among clades unrelated to presence of the region itself.

Itoh and colleagues observed variable colonization success in PBE, in which infection rates varied from 0 to 100%, and we found that this was unrelated to the presence of the contiguous genomic region. SBE species were able to colonize the M4 densely, and 8/9 species possessed the contiguous region. Burkholderia glathei was the only SBE found lacking the region, but it possessed all 40 genes and had a 100% infection rate. Although we cannot rule out the possibility that possession of the contiguous symbiosis-associated region contributes to establishment in the M4 organ, it is neither necessary nor sufficient for host colonization. (One caveat to this finding is that the strains used by Itoh et al. [15] are not necessarily identical to the ones whose genomes were sequenced, and it is possible the genomic region could be lost or gained among different strains within the same species.)

Contrary to our expectations, some *Burkholderiaceae* species lacking the contiguous genomic region could still colonize the M4. For example, *Pandoraea* lacks the region but can still colonize the M4 crypts, resulting in improved insect survival, development time, and mass at adulthood, though to a lesser degree than members of the SBE and PBE *Burkholderia* clades (15). Interestingly, *Pandoraea* does not fill the M4 crypts as thoroughly as SBE species (15). Regardless of the degree in which the M4 crypts are filled, successful colonization directly benefits the host because the insect digests the *Burkholderia* cells as a source of nutrition. Therefore, when the host does not have a symbiont, often the host does not survive to adulthood, or if it does survive to adulthood, it has severe developmental delays, infertility, or other health defects. In addition, evidence suggests that the M4 organ is a stressful environment for *Burkholderia*, indicating that the genes found within the genomic region may potentially promote better or denser colonization of the M4 crypts or improve symbiont survival in the hostile environment of the host’s gut, perhaps by providing resistance to the host’s antimicrobial peptide defenses (19). Other genes located outside the putative island are known to be important for M4 colonization, including those encoding stress tolerance factors, biofilm formation, and flagellar motility (12, 18, 42–44). Genes with similar functionality are required by *Snodgrassella* to colonize the bee gut (41), suggesting that these functions may be generally important for symbiont colonization of the insect gut.

### Future directions.

Future experiments that correlate presence of the contiguous region (or genes found within the region) not only with M4 colonization success but also with physiological effects on the insect and, ultimately, insect fitness are warranted. Gene knockout studies will help determine the function of particular genes from the genomic region; such studies have been performed at the whole-genome level (18, 42, 43), but focusing on genes in the symbiosis-associated region may be more likely to identify pathways and functions that underpin the bug-*Burkholderia* symbiosis. Experimental addition of the genomic region to *Burkholderiaceae* species that lack it could also help to decipher functionality. The region’s genes could be excised from any of the insect symbionts and synthesized into a plasmid for insertion into new potential hosts. Sequencing the genomes of vertically transmitted *Burkholderia* symbionts, such as those found in chinch bugs (Blissidae) (45, 46), could also be enlightening. In vertically transmitted symbioses, evolutionary genome degradation purges microbial genes that do not contribute to symbiosis (47). Comparison of vertically transmitted *Burkholderia* might therefore help identify the essential genes located within the genomic region.

### Conclusions.

Through this study, we found that the putative bug-*Burkholderia* symbiotic island may be not a genomic island but instead a genomic region containing numerous symbiosis-associated genes. The contiguous arrangement is a relatively recent feature which might play a role in the bug association, as the contiguous 40-gene region is largely conserved across the insect-associated SBE and PBE *Burkholderia*. Exploration of the function and evolution of these 40 genes and the significance of their contiguous arrangement will provide a better understanding of how these genes are associated with hemipteran symbiosis while also yielding a more generalized understanding of the genetic foundations of symbioses that will have broad relevance for the genomics of host-microbe interactions.

## MATERIALS AND METHODS

### Insect collection and isolation of symbiont DNA.

To increase the diversity and number of available symbiont genomes from symbiotic *Burkholderia*, we obtained seven symbiont isolates from true bugs, and one from soil near true bugs, in North America. Specifically, strains Lep1A1 and Lep1P3 were isolated from western leaf-footed bugs (Leptoglossus zonatus, Coreidae) collected in California. SL2Y3 and TF1N1 were isolated from spined stilt bugs (Jalysus wickhami, Berytidae) collected in Arizona and North Carolina, respectively. Sq4a and A33_M4_a were isolated from squash bugs (Anasa tristis, Coreidae) captured in Georgia. BHJ32i was obtained from a broad-headed bug (Alydus tomentosus, Alydidae) collected in Georgia. SMT4a was isolated from soil collected in Georgia at a site where broad-headed bugs (*Alydus* sp.) were present (Table 1).

To isolate symbionts from *L. zonatus* and *J. wickhami*, individual insects were sacrificed and simultaneously surface sterilized by submerging them in 95% ethanol for 2 min. Each insect was dissected and the M4 gut region (which hosts *Burkholderia*) was carefully excised. The M4 was rinsed in sterile water to remove any bacteria from the surface of the organ; then it was submerged in yeast-glucose (YG) broth at room temperature for 24 to 48 h, with shaking at 270 rpm. (This organ culture step increases the success of isolations, presumably by allowing the *Burkholderia* to acclimate more gradually to a free-living mode of life [48]). The M4 was then removed from the broth, homogenized, and plated on YG agar. Individual colonies were streaked to isolation and subjected to diagnostic PCR to confirm that the isolate was indeed *Burkholderia*. Original isolates were stored in 40% glycerol at −80°C. To obtain DNA for genome sequencing, frozen stocks were streaked on YG agar and a single colony was inoculated into broth for overnight incubation. DNA was extracted from 1 mL of the resulting culture using the Wizard Genomic DNA purification kit (A1120; Promega, Madison, WI).

Methods for isolation and DNA extraction of *Burkholderia* from *A. tristis* and *A. tomentosus* were similar to those described above; they have been described in detail by Acevedo et al. (27) and Garcia et al. (49). Isolation of SMT4a from soil has been described by Garcia et al. (49).

### Sequencing.

Genomic DNA was sequenced by SNPsaurus (Eugene, OR) on an Illumina HiSeq 4000 (Illumina, San Diego, CA), following their standard workflow for library preparation and read trimming. A Nextera tagmentation kit (Illumina) was used for library generation, followed by paired-end 150-bp sequencing at greater than 60× coverage for each of the eight isolates. Adaptors were trimmed from the reads with BBDuk (50).

To obtain long reads for scaffolding, we also sequenced the DNA on an Oxford Nanopore MinION (Oxford Nanopore, England, UK). We prepared 1 μg of DNA per isolate using the rapid sequencing kit (RAD004; Oxford Nanopore), and all DNA was unsheared prior to library preparation. We ran four isolates per flow cell sequentially. Although the flow cell was washed after each isolate, we barcoded each with a rapid barcoding kit (RBK004; Oxford Nanopore) to guard against cross-contamination. Reads were called during sequencing using Guppy v2.0.10, and only the reads which passed the initial quality check during the run (and which were therefore placed into the “fast5_pass” folder) were used (51).

### Genome assembly and annotation.

Sequencing data (Illumina and Nanopore) were uploaded to Cyverse (formerly iPlant Collaborative) (52), where the NanoDJ application (53) was used to assemble the genomes (raw reads under SRA BioProject PRJNA765207). Unicycler v0.4.8-beta (54) and SPAdes v3.13.0 (55) were used to perform hybrid assemblies utilizing both Nanopore and Illumina reads. Genomes were annotated with DFAST v1.2.4 (56) and visualized using Geneious Prime v2020.2.4. Chromosome and plasmid names were assigned using megablast and comparing each of the eight genomes to a reference genome (*Burkholderia insecticola* [accession no. GCA_000402035.1]). Default parameters were used for all analyses unless otherwise specified.

### Species identification and chromosome comparison.

We used an ANI calculator (http://enve-omics.ce.gatech.edu/ani/) (57) using the default settings to determine average nucleotide identity between our eight genomes and their closest related species on the generated phylogeny to determine if the members within their terminal clades were the same species (58).

### Phylogenetic analysis.

We constructed a phylogenetic tree using our 8 assembled genomes plus 204 genomes belonging to the family *Burkholderiaceae* (all species that were available except for *Polynucleobacter* species; see below) from the National Center for Biotechnology Information (NCBI) GenBank database and one genome from *Alcaligenaceae* (Bordetella bronchiseptica) as an outgroup (Table S1). The 17 species from the *Polynucleobacter* genus were dropped from the analysis because they were too dissimilar to the rest of the species in *Burkholderiaceae*, causing errors in the construction of the phylogeny. (Incidentally, this suggests that *Polynucleobacter* may be taxonomically misplaced.)

The phylogeny was constructed using Realphy (59) with the gap threshold set to “0.1,” and Burkholderia cepacia used as the reference genome. This program used Bowtie2 v2.0.0 (60) to map each genome to the reference genome. The output was then used in RAxML v8.2.10 (61), where the maximum likelihood phylogenetic tree was inferred with the GTR+Gamma model of nucleotide substitution. Node support values were calculated using 500 rounds of rapid bootstrapping. Phylogenies were visualized with ape v5.4-1 (62).

We used ancestral state reconstruction to determine when the contiguous genomic region most likely arose in *Burkholderiaceae*. We generated two discrete models, including an equal-rates model and an all-rates-different matrix model (function = “ace”; package = “ape”) (62). To find the model that best explained these data, we performed a chi-square test comparing the equal-rates model and all-rates-different matrix model (function = “pchisq”) (63). The all-rates-different matrix model was selected based on the results of the chi-square test (*P* value ≤ 0.01).

### Structure, order, and content of the symbiosis-associated region across the *Burkholderiaceae*.

To initially locate the genomic region within the various genomes, we scanned for the PQQ gene cluster using a megablast search via Geneious Prime and followed this with a BLASTX to identify any PQQ gene clusters that were missed in the initial search. The chromosome (or plasmid) in which the PQQ genes were located was excised from the genome, and all chromosomes were aligned using Mauve v1.1.13 (64). Because the order of the genes in the region (if present) could not be inferred from assemblies in which the island was split across two or more contigs, we only used fully assembled genomes and contig assemblies that contained the entire putative island on a single contig for this analysis. We compared structure, gene order, and gene contents of the genomic region across the *Burkholderiaceae* family. When discrepancies in gene annotations occurred (e.g., differences between the predicted identity or function at the same locus in different genomes), we compared the genes to the NCBI database using BLASTX to determine if the proteins were truly different or orthologous but inconsistently named across species. We used A33_M4_a (SBE) and Paraburkholderia phymatum (PBE) as the reference genomes for protein comparisons on NCBI. Sequences flanking the contiguous region were BLAST searched against the NCBI database to identify if they aligned with any known transposable elements, and the annotations within these regions were also observed to gauge whether annotated transposons were detected by the annotation software. To detect phage elements in genomes, sequences were tested using PHASTER (65, 66). The chromosome (or contig) that contained the island was submitted to the server and scanned for the presences of phage elements.

### Identification of orthologous genes.

To identify if species without the contiguous genomic region possessed orthologous genes, a BLASTX was performed via NCBI using all 40 genes from A33_M4_a. First, we searched broadly across all bacteria, excluding *Burkholderiaceae*, to identify whether the genes were ancestral and present in other bacterial families, predominately focusing on those within the *Burkholderiales* order. Next, we used BLASTX to search for each of the 40 genes found in the genomic region within all 176 *Burkholderiaceae* genomes that did not possess the contiguous genomic region. If the search result had an E value of ≤e^−20^ and a query coverage of ≥70%, it was considered a strong ortholog candidate. We also considered proteins to be potential orthologs is they had two of the three following criteria: the query protein had the same name as the searched protein, an E value of ≤e^−3^, or a query coverage of ≥30%.

### Phylogenetic analysis of the symbiosis-associated region.

Genomic islands are typically characterized by instances of horizontal gene transfer. To test for horizontal gene transfer within *Burkholderiaceae*, we constructed phylogenies for four genes from the contiguous genomic region that were previously shown to be upregulated during symbiosis and compared these to a whole-genome phylogeny consisting of the 53 species with the contiguous region (with the contiguous region itself excised for this comparison) (18). The four selected genes were those encoding amino acid ABC transporter substrate-binding protein, coenzyme PQQ synthesis protein E, formylmethanofuran dehydrogenase subunit A, and sigma-54-dependent Fis family transcriptional regulator. Genes were aligned using MAFFT v7.450 (67) with the default settings and phylogenies were created with RAxML, using the same conditions as those described in the phylogenetic analysis section above.

### Testing necessity of the genomic region for bug colonization.

To test whether the presence of the genomic region is associated with a microbe’s ability to colonize the M4 organ, we took advantage of a previously published data set on the M4 infection rate of different *Burkholderiaceae* species. Itoh et al. (15) exposed 2nd-instar *R. pedestris* nymphs to different potential colonizing bacteria within *Burkholderiaceae*. After the nymphs molted to 3rd instar, the authors performed diagnostic PCR on 10 individuals infected with each of the symbiont species to determine the overall infection rate.

We noted the presence or absence of the contiguous region for 34 of the *Burkholderiaceae* species tested by Itoh et al., spanning the *Burkholderia* clades BCC&P (6 species), PBE (12), and SBE (9) as well as the closely related genera *Chitinimonas* (1), *Cupriavidus* (1), *Pandoraea* (2), *Ralstonia* (1), *Robbsia* (1), and *Trinickia* (1). We binned these into species that contained the contiguous genomic region and those without the region (Table S4). To determine whether bacterial species with and without the region differed in the ability to colonize the M4, we performed a binomial logistic regression with insect colonization success as the dependent variable and presence of the contiguous region as a fixed effect. Since Itoh et al. (15) found that M4 colonization success varies among bacterial clades, we also modeled colonization as a function of both genomic region presence and symbiont clade membership. We note that the strains tested by Itoh et al. are likely different strains than those found on GenBank, and so the genomic region’s structure may differ, but we based our analysis on the assumption that the presence and structure of the region will not vary significantly between strains of the same species. All statistical analyses were conducted in R v3.6.0.

### Data availability.

The genome sequences for A33_M4_a, BHJ32i, Lep1A1, Lep1P3, SL2Y3, SMT4a, Sq4a, and TF1N1 were deposited in GenBank with accession numbers listed in Table 1 and Table S1. The raw Illumina and Nanopore reads have been uploaded to SRA under BioProject PRJNA765207.
