# Supplementary material for: Prevalence of an Insect-Associated Genomic Region in Environmentally Acquired Burkholderiaceae Symbionts
Source: Appl Environ Microbiol. 2022 Apr 18;88(9):e02502-21. doi: 10.1128/aem.02502-21 (PMC9088363; doi:10.1128/aem.02502-21)
Supplement: Supplemental file 1 — Fig. S1. Download aem.02502-21-s0001.pdf, PDF file, 0.5 MB [file aem.02502-21-s0001.pdf]

Supporting information

Fig S1. Phylogenetic comparison of four upregulated genes from the contiguous genomic region. Phylogenetic arrangement of species containing the contiguous genomic region (with the region removed) and select upregulated genes from all species that had the contiguous region. Trees include the whole genome phylogeny (A), amino acid ABC transporter substrate-binding protein (B), formylmethanofuran dehydrogenase subunit A (C), coenzyme PQQ synthesis protein E (D), and sigma-54-dependent Fis family transcriptional regulator (E). Trees were rooted with species A33_M4_a for consistency as no outgroup was available for the contiguous region**.** Species names in the phylogenies were color coded based on the order listed in the whole genome phylogeny to improve visualizing incongruencies. The black asterisks indicate potential horizontal transfer events. Node support values were calculated using rapid bootstrapping and those with less than 50% support have been collapsed. Scale bar indicates substitutions per site.

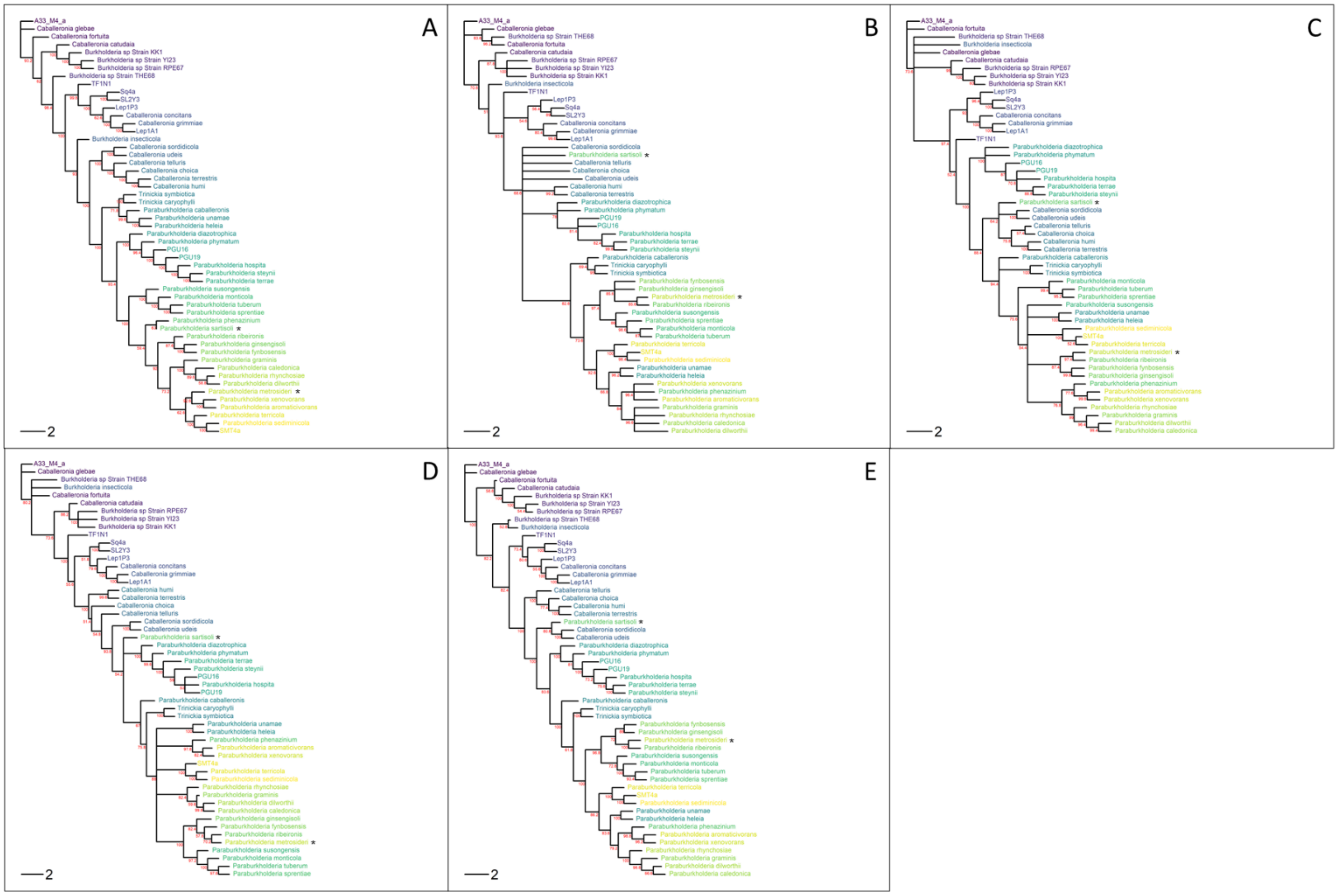
A
A33_M4_a
Caballeronia glebae
Caballeronia fortuita
Caballeronia catudaia
Burkholderia sp Strain KK1
Burkholderia sp Strain YI23
Burkholderia sp Strain RPE67
Burkholderia sp Strain THE68
TF1N1
Sq4a
SL2Y3
Lep1P3
Caballeronia concitans
Caballeronia grimmiae
Lep1A1
Burkholderia insecticola
Caballeronia sordidicola
Caballeronia udeis
Caballeronia telluris
Caballeronia choica
Caballeronia terrestris
Caballeronia humi
Trinickia symbiotica
Trinickia caryophylli
Paraburkholderia caballeronis
Paraburkholderia unamae
Paraburkholderia heleia
Paraburkholderia diazotrophica
Paraburkholderia phymatum
PGU16
PGU19
Paraburkholderia hospita
Paraburkholderia steynii
Paraburkholderia terrae
Paraburkholderia susongensis
Paraburkholderia monticola
Paraburkholderia tuberum
Paraburkholderia sprentiae
Paraburkholderia phenazinium
Paraburkholderia sartisoli *
Paraburkholderia ribeironis
Paraburkholderia ginsengisoli
Paraburkholderia fynbosensis
Paraburkholderia graminis
Paraburkholderia caledonica
Paraburkholderia rhynchosiae
Paraburkholderia dilworthii
Paraburkholderia metrosideri *
Paraburkholderia xenovorans
Paraburkholderia aromaticivorans
Paraburkholderia terricola
Paraburkholderia sediminicola
SMT4a
2
B
A33_M4_a
Caballeronia glebae
Burkholderia sp Strain THE68
Caballeronia fortuita
Caballeronia catudaia
Burkholderia sp Strain RPE67
Burkholderia sp Strain YI23
Burkholderia sp Strain KK1
Burkholderia insecticola
TF1N1
Lep1P3
Sq4a
SL2Y3
Caballeronia concitans
Caballeronia grimmiae
Lep1A1
Caballeronia sordidicola
Paraburkholderia sartisoli *
Caballeronia telluris
Caballeronia choica
Caballeronia udeis
Caballeronia humi
Caballeronia terrestris
Paraburkholderia diazotrophica
Paraburkholderia phymatum
PGU19
PGU16
Paraburkholderia hospita
Paraburkholderia terrae
Paraburkholderia steynii
Paraburkholderia caballeronis
Trinickia caryophylli
Trinickia symbiotica
Paraburkholderia fynbosensis
Paraburkholderia ginsengisoli
Paraburkholderia metrosideri *
Paraburkholderia ribeironis
Paraburkholderia susongensis
Paraburkholderia sprentiae
Paraburkholderia monticola
Paraburkholderia tuberum
Paraburkholderia terricola
SMT4a
Paraburkholderia sediminicola
Paraburkholderia unamae
Paraburkholderia heleia
Paraburkholderia xenovorans
Paraburkholderia phenazinium
Paraburkholderia aromaticivorans
Paraburkholderia graminis
Paraburkholderia rhynchosiae
Paraburkholderia caledonica
Paraburkholderia dilworthii
2
C
A33_M4_a
Caballeronia fortuita
Burkholderia sp Strain THE68
Burkholderia insecticola
Caballeronia glebae
Caballeronia catudaia
Burkholderia sp Strain RPE67
Burkholderia sp Strain YI23
Burkholderia sp Strain KK1
Lep1P3
Sq4a
SL2Y3
Caballeronia concitans
Caballeronia grimmiae
Lep1A1
TF1N1
Paraburkholderia diazotrophica
Paraburkholderia phymatum
PGU16
PGU19
Paraburkholderia hospita
Paraburkholderia terrae
Paraburkholderia steynii
Paraburkholderia sartisoli *
Caballeronia sordidicola
Caballeronia udeis
Caballeronia telluris
Caballeronia choica
Caballeronia humi
Caballeronia terrestris
Paraburkholderia caballeronis
Trinickia caryophylli
Trinickia symbiotica
Paraburkholderia monticola
Paraburkholderia tuberum
Paraburkholderia sprentiae
Paraburkholderia susongensis
Paraburkholderia unamae
Paraburkholderia heleia
Paraburkholderia sediminicola
SMT4a
Paraburkholderia terricola
Paraburkholderia metrosideri *
Paraburkholderia ribeironis
Paraburkholderia fynbosensis
Paraburkholderia ginsengisoli
Paraburkholderia phenazinium
Paraburkholderia aromaticivorans
Paraburkholderia xenovorans
Paraburkholderia rhynchosiae
Paraburkholderia graminis
Paraburkholderia dilworthii
Paraburkholderia caledonica
2
D
A33_M4_a
Caballeronia glebae
Burkholderia sp Strain THE68
Burkholderia insecticola
Caballeronia fortuita
Caballeronia catudaia
Burkholderia sp Strain RPE67
Burkholderia sp Strain YI23
Burkholderia sp Strain KK1
TF1N1
Sq4a
SL2Y3
Lep1P3
Caballeronia concitans
Caballeronia grimmiae
Lep1A1
Caballeronia humi
Caballeronia terrestris
Caballeronia choica
Caballeronia telluris
Caballeronia sordidicola
Caballeronia udeis
Paraburkholderia sartisoli *
Paraburkholderia diazotrophica
Paraburkholderia phymatum
Paraburkholderia terrae
Paraburkholderia steynii
PGU16
Paraburkholderia hospita
PGU19
Paraburkholderia caballeronis
Trinickia caryophylli
Trinickia symbiotica
Paraburkholderia unamae
Paraburkholderia heleia
Paraburkholderia phenazinium
Paraburkholderia aromaticivorans
Paraburkholderia xenovorans
SMT4a
Paraburkholderia terricola
Paraburkholderia sediminicola
Paraburkholderia rhynchosiae
Paraburkholderia graminis
Paraburkholderia dilworthii
Paraburkholderia caledonica
Paraburkholderia ginsengisoli
Paraburkholderia fynbosensis
Paraburkholderia ribeironis
Paraburkholderia metrosideri *
Paraburkholderia susongensis
Paraburkholderia monticola
Paraburkholderia tuberum
Paraburkholderia sprentiae
2
E
A33_M4_a
Caballeronia glebae
Caballeronia fortuita
Caballeronia catudaia
Burkholderia sp Strain KK1
Burkholderia sp Strain YI23
Burkholderia sp Strain RPE67
Burkholderia sp Strain THE68
Burkholderia insecticola
TF1N1
Sq4a
SL2Y3
Lep1P3
Caballeronia concitans
Caballeronia grimmiae
Lep1A1
Caballeronia telluris
Caballeronia choica
Caballeronia humi
Caballeronia terrestris
Paraburkholderia sartisoli *
Caballeronia sordidicola
Caballeronia udeis
Paraburkholderia diazotrophica
Paraburkholderia phymatum
PGU16
PGU19
Paraburkholderia hospita
Paraburkholderia terrae
Paraburkholderia steynii
Paraburkholderia caballeronis
Trinickia caryophylli
Trinickia symbiotica
Paraburkholderia fynbosensis
Paraburkholderia ginsengisoli
Paraburkholderia metrosideri *
Paraburkholderia ribeironis
Paraburkholderia susongensis
Paraburkholderia monticola
Paraburkholderia tuberum
Paraburkholderia sprentiae
Paraburkholderia terricola
SMT4a
Paraburkholderia sediminicola
Paraburkholderia unamae
Paraburkholderia heleia
Paraburkholderia phenazinium
Paraburkholderia aromaticivorans
Paraburkholderia xenovorans
Paraburkholderia rhynchosiae
Paraburkholderia graminis
Paraburkholderia dilworthii
Paraburkholderia caledonica
2
